# How have the European central bank’s monetary policies been affecting financial markets in CEE-3 countries?

**DOI:** 10.1007/s40822-020-00160-3

**Published:** 2020-12-02

**Authors:** Wojciech Grabowski, Ewa Stawasz-Grabowska

**Affiliations:** 1 Department of Econometric Models and Forecasts, Faculty of Economics and Sociology, University of Lodz, Rewolucji 1905 r. No. 37/39, 90-214 Lodz, Poland; 2 Department of International Economics, Faculty of Economics and Sociology, University of Lodz, Rewolucji 1905 r. No. 41, 90-214 Lodz, Poland

**Keywords:** Central and eastern europe, ECB’s monetary policy, Spillover effects, AGDCC-GARCH model, C58, F36, E58, G12, G15

## Abstract

This paper aims to contribute to the growing pool of literature on the spillover effects of the European Central Bank’s (un)conventional monetary policies on the exchange rate, sovereign bond and equity markets of the Czech Republic, Hungary and Poland (CEE-3 countries), which are collectively known as the CEE-3 countries. The study is conducted using daily data from January 2010 to September 2019. Our results indicate that the financial markets of the CEE-3 countries have been strongly influenced by the nonstandard measures enacted by the European Central Bank, particularly those involving purchases of euro-area sovereign debt. The strongest spillover effects were identified for the Securities Markets Program, while the effects from the Outright Monetary Transactions program turned out to be the most durable. At the same time, the financial markets of the CEE-3 countries were found to have been largely unaffected by interest rate changes enacted by the European Central Bank.

## Introduction

During the global financial crisis, which, in the euro area, took the form of a debt crisis in some of the European Union member states, the European Central Bank (ECB) launched a number of unconventional monetary policy measures. Some of them were maintained after 2012 despite the already clearly marked decline in tensions in the sovereign bond market of the European Economic and Monetary Union (EMU). Moreover, in the following years, the ECB undertook further initiatives of this type, the last of which was introduced in 2019.^1^

In this paper, we sought to gauge the impact of monetary policy measures, particularly nonstandard ones, introduced by the ECB from January 2010 to September 2019 on financial markets of the so-called CEE-3 countries—the Czech Republic, Hungary and Poland. Specifically, our goals in this paper were (1) to assess financial market responses to the ECB’s announcements of monetary policy measures, with a particular focus on the exchange rate, sovereign bond and equity markets of the CEE-3 countries; (2) to examine the durability of the spillover effects from a particular category of the ECB’s (un)conventional measures; and (3) to evaluate the impact of the ECB’s actions on financial market volatility in the CEE-3 countries.

To achieve these goals, we created a list of the ECB’s monetary policy announcements, primarily using press releases available on the ECB’s website. In the case of nonstandard monetary policy measures, we included the dates of their announcements; disclosures of their technical details; and the dates of their launch and end, if applicable. We estimated the parameters of multivariate GARCH models including variables associated with the ECB’s measures. We also estimated the parameters of models explaining market volatilities to assess whether nonstandard ECB measures decrease or increase the level of uncertainty in financial markets.

Our paper can be linked to at least three strands of research. First, in most general terms, this paper adds to the ample literature on the effects of unconventional monetary policy of the ECB (De Grauwe and Ji 2014; Falagiarda and Reitz 2015; Kilponen et al. 2015; Dewachter et al. 2016; Boysen-Hogrefe 2017; Afonso et al. 2018; De Santis 2020). The literature suggests largely that the ECB’s nonstandard measures triggered substantial financial market responses, especially in the sovereign bond segment, although it predominantly concentrates on the euro-area countries.

Second, this paper is related to the literature on the global effects of unconventional monetary policy measures of the major central banks. This research is dominated by analyses devoted to the impact of the U.S. Federal Reserve’s policies, which seems to reflect the key role of the United States in the global economy (Bauer and Neely 2014; Chen et al. 2014, 2016; Kiendrebeogo 2016; Borrallo et al. 2016). The overall picture from this literature is that U.S. monetary policy shocks exerted international spillovers, which turned out to be particularly strong in emerging market economies characterized by weaker macroeconomic fundamentals, higher dollarization of assets and liabilities and stronger commercial and financial ties with the United States. At the same time, prior studies, which were aimed at comparing the cross-border impacts of U.S. and euro-area monetary policies, point to larger and more persistent effects of the Federal Reserve’s actions and regionally limited scope of influence of the ECB’s measures (Chen et al. 2017; Apostolou and Beirne 2019). Hence, the vast majority of existing studies dealing with the spillover effects from the ECB’s monetary policy have focused on responses of the Central and Eastern European (CEE) economies that are more linked to the euro area.

Notably, some of those studies have concentrated on the economic impact of the ECB’s policy actions and usually employ VAR estimation techniques. In general, they provide evidence that the ECB’s expansionary monetary policy in recent years exerted strong effects on the real economy of the CEE countries in the form of increases in inflationary pressure together with growth in the gross domestic product or industrial production (Horvath and Voslarova 2016; Potjagailo 2017; Moder 2019; Feldkircher et al. 2020). At the same time, the spillover effects of the ECB’s conventional measures on the macroeconomic developments have been found to be stronger than those of unconventional measures (Hajek and Horvath 2018; Babecká Kucharčuková et al. 2016).

Other studies devoted to assessing the sensitivity of the CEE economies focused on the financial market impact of monetary policy announcements. Generally, this literature concluded that the announcements related to the ECB’s measures involving the purchases of sovereign bonds triggered a broad-based appreciation of CEE currencies vis-à-vis the euro and a moderate compression of long-term sovereign bond yields. At the same time, individual evaluations point to a variable magnitude and direction of the impact of those announcements on stock market indices of CEE countries (Falagiarda et al. 2015; Ciarlone and Colabella 2016; Varghese and Zhang 2018). Furthermore, the existing literature is inconclusive with regard to stating the role played by individual asset purchase programs. For example, Falagiarda et al. (2015) find that, among the ECB’s measures involving the purchase of sovereign bonds, the Securities Markets Program (SMP) announcements had the strongest impact on financial assets in the Czech Republic, Hungary, Poland and Romania. At the same time, the authors identified weak effects in the case of the Outright Monetary Transactions (OMT) announcements and limited in the case of the Public Sector Purchase Program (PSPP) announcements. Fratzscher et al. (2016) identified positive effects from the SMP and OMT announcements in the form of declining sovereign bond yields and increasing bank equity prices among a group of emerging E.U. countries. Finally, Georgiadis and Gräb (2016) have offered evidence for weaker financial market responses in non–euro-area E.U. countries to the OMT and SMP announcements as compared with those related to the Asset Purchase Program (APP), particularly in the foreign-exchange market segment.

Third, this paper adds to the available literature on linkages among financial markets in CEE countries with the use of multivariate GARCH models (Syllignakis and Kouretas 2011; Grabowski 2019). However, in previous papers, the role of monetary policy measures was not typically exploited. Adding additional groups of variables, which affect rates of return on stock indexes and exchange rates as well as changes in sovereign bond yields, should result in more reliable estimates of parameters that reflect linkages among markets.

Notwithstanding a growing number of empirical studies on the international spillovers of the ECB’s monetary policy, we still believe we can contribute to the existing literature. In particular, we include the ECB’s most recent initiatives in this research, which have not been accounted for in previous analyses. This enables us to compare the nature of spillovers in turbulent and relatively tranquil periods. Moreover, as compared with other authors using the event study methodology, we employed a larger variety of window lengths, which enabled us to draw more accurate conclusions about spillover effects, especially in terms of their timing of occurrence and durability. For example, Kilponen et al. (2015) considered two- and three-day event windows, while Altavilla et al. (2016) considered event windows of lengths ranging from one to five days. Accordingly, we considered one-, three-, six- and 11-day windows, which makes it possible for us to compare the durability of consecutive measures. The authors of previous studies focused on the impact of the ECB’s (un)conventional monetary policy measures on changes in the prices of financial instruments (Falagiarda and Reitz 2015; Grabowski and Stawasz-Grabowska 2019); similarly, we analyzed the effects of the ECB’s measures on the level of uncertainty in CEE-3 financial markets. The analysis of the impact of measures on uncertainty provides additional knowledge about the efficiency of decisions of the ECB as well as those of the national banks of Poland, Hungary and the Czech Republic.

The paper is structured as follows: Sect. 1 is the introduction. Section 2 describes the data and explains the methodology used. Section 3 discusses the empirical results. Section 4 concludes the paper.

## Variables used in the empirical study—econometric model

### Definitions and names of variables

The study was conducted involving CEE-3 countries with the sample period running from January 2010 until September 2019. The lower boundary relates to the year of the outbreak of the euro-area sovereign debt crisis. The choice of the upper boundary was conditional on the availability of data at the time of the study. The frequency of data is daily (five-day week). The set of dependent variables consists of the following:

- Daily rates of return on exchange rates (EUR/CZK, EUR/HUF, EUR/PLN).
- Daily changes of 10-year sovereign bond yields of the Czech Republic, Hungary and Poland.
- Daily rates of return on stock market indexes in the analyzed countries (PX, BUX, WIG stand for the main stock exchange indices from the Czech Republic, Hungary and Poland respectively).

The respective time series were obtained from the Thomson Reuters Eikon and Bloomberg databases. We considered differences between the price at day *t* and that at day *t − 1*. This approach is not the same as that proposed by Altavilla et al. (2019), who considered intra-daily changes in prices of financial instruments in press releases and conference windows.

The set of explanatory variables is dominated by the binary variables associated with interest-rate changes and unconventional monetary policy measures launched by the ECB in the research period. Moreover, as control variables, we included the following four categories:

- Monetary policy measures undertaken by the national banks of the Czech Republic, Hungary and Poland.
- Daily rates of return on DAX and daily changes of 10-year German sovereign bond yields.
- VSTOXX index measuring the volatility in European equity markets.
- Macroeconomic surprises for some key macroeconomic indicators of the CEE-3 countries.

Below, variables associated with the ECB measures will be covered in greater detail.

As already indicated, in the period January 2010 to September 2019, the ECB introduced a variety of initiatives so as to ease its monetary policy stance. Chronologically, they are as follows:

- Measures introduced within the framework of the enhanced credit support aimed at averting a major credit crunch in the euro area (second covered bond purchase program [CBPP2] and two three-year, longer-term refinancing operations [3Y LTRO]).
- Two programs allowing the ECB to buy sovereign bonds of the euro area countries in the secondary market—SMP and OMT. Under the SMP, which was launched in May 2010, the ECB acquired around €220 billion of Greek, Irish, Italian, Portuguese and Spanish government bonds. The program was terminated with the announcement of the OMT, which occurred in September 2012. The OMT, which has never been applied, allowed for unlimited purchases of government bonds and therefore was widely identified with the ECB entering the role of a lender of last resort for euro-area sovereigns (De Grauwe and Ji 2014; Winkler 2015).
- A package of measures initiated in mid-2014 amidst risks of a subdued inflation outlook and weak growth. The package, whose objective was to support the monetary policy transmission mechanism and facilitate credit provision to the real economy and the euro-area recovery, included targeted longer-term refinancing operations (TLTRO) and the APP consisting of the corporate-sector purchase program (CSPP), public-sector purchase program (PSPP), asset-backed securities purchase program (ABSPP) and third covered-bond purchase program (CBPP3). The APP expired in December 2018; however, the ECB decided to restart net purchases in September 2019 given the background of muted inflationary pressure and downward revisions to the outlook for euro-area economic growth.

We also account for changes to the ECB’s key interest rates. In the empirical investigation, we consider binary variables associated with decisions of the ECB.

A variable adopts a value of 1 at the day of decision and that of 0 otherwise. The names and definitions of variables associated with the ECB’s measures are presented in Table 1.

**Table 1:** Variables associated with ECB measures

Measure		Date	Event	Variable
Interest rate policy		3 November 2011, 8 December 2011, 5 July 2012, 2 May 2013, 7 November 2013, 5 June 2014, 4 September 2014, 3 December 2015, 10 March 2016, 12 September 2019	ECB decides to lower its key interest rates	*INT-RATE-DOWN_ECB*
		7 April 2011, 7 July 2011	ECB decides to increase its key interest rates	*INT-RATE-UP_ECB*
3Y LTRO		8 December 2011	ECB announces two LTROs with a maturity of 3 years	*LTRO_ANNOUNCEMENT*
		21 December 2011	The first 3Y LTRO is allotted	*LTRO(1)_ALLOTMENT*
		22 December 2011	The first 3Y LTRO is settled	*LTRO(1)_SETTLEMENT*
		29 February 2012	The second 3Y LTRO is allotted	*LTRO(2)_ALLOTMENT*
		1 March 2012	The second 3Y LTRO is settled	*LTRO(2)_SETTLEMENT*
SMP		10 May 2010	ECB announces SMP	*SMP_ANNOUNCEMENT*
CBPP2		6 October 2011	ECB announces CBPP2	*CBPP2_ANNOUNCEMENT*
		3 November 2011	ECB announces details of CBPP2	*CBPP2_DETAILS*
OMT		26 Jul y2012	Mario Draghi gives ‘whatever it takes’ speech	*OMT_ANNOUNCEMENT*
		6 September 2012	ECB announces details of OMTs	*OMT_DETAILS*
APP	CBPP3 and ABSPP	4 September 2014	ECB announces CBPP3 and ABSPP	*CBPP3-ABSPP_ANNOUNCEMENT*
		2 October 2014	ECB announces details of CBPP3 and ABSPP	*CBPP3-ABSPP_DETAILS*
		20 October 2014	ECB starts to buy covered bonds under CBPP3	*CBPP3_START*
		21 November 2014	ECB starts to buy asset-backed securities under ABSPP	*ABSPP_START*
	PSPP	22 January 2015	ECB announces PSPP	*PSPP_ANNOUNCEMENT*
		9 March 2015	ECB starts to buy public sector securities under PSPP	*PSPP_START*
	CSPP	10 March 2016	ECB announces CSPP	*CSPP_ANNOUNCEMENT*
		21 April 2016	ECB announces details of CSPP	*CSPP_DETAILS*
		8 June 2016	ECB starts to buy corporate sector bonds under CSPP	*CSPP_START*
	APP	3 September 2015, 3 December 2015, 10 March 2016, 8 December 2016, 26 October 2017, 14 June 2018	ECB announces APP extensions in terms of technical modalities, size or duration	*APP-EXTENSION_ ANNOUNCEMENT*
		13 December 2018	ECB announces the end of net purchases under APP in December 2018	*APP-END_ ANNOUNCEMENT*
		12 September 2019	ECB announces the restart of net purchases under APP	*APP-RESTART_ ANNOUNCEMENT*
TLTRO I		5 June 2014	ECB announces the first series of TLTROs	*TLTRO(1)_ANNOUNCEMENT*
		24 September 2014, 17 December 2014, 25 March 2015, 24 June 2015, 30 September 2015, 16 December 2015, 30 March 2016, 29 June 2016	The consecutive operations under TLTRO I are settled	*TLTRO(1)_SETTLEMENT*
TLTRO II		10 March 2016	ECB announces the second series of TLTROs	*TLTRO(2)_ANNOUNCEMENT*
		29 June 2016, 28 September 2016, 21 December 2016	The consecutive operations under TLTRO II are settled	*TLTRO(2)_SETTLEMENT*
TLTRO III		7 March 2019	ECB announces the third series of TLTROs	*TLTRO(3)_ANNOUNCEMENT*

Source: Authors’ own compilation based on the ECB’s press releases

*The list of events is constructed as an extension of the one used in the study of *Grabowski and Stawasz-Grabowska (2019)

Apart from variables associated with unconventional measures of the ECB, control variables associated with domestic monetary policy measures in the CEE-3 countries, the performance of financial markets in Germany, volatility in European equity markets and macroeconomic surprises are used as explanatory ones. Since we are herein concentrating on the impact of the ECB’s measures, we did not provide names and definitions of control variables in the main text. In Appendix 1, the construction of these variables is described.

### Methodology

We analyzed the impact of the ECB’s monetary policy measures and the variables from the other four explanatory categories on changes in prices of the three financial instruments on the day of an announcement as well as in windows of different lengths (e.g., one-, three-, six-, and 11-day). Therefore, we define the names of variables on the basis of window lengths. For example, the variable *PSPP_START-W3*_*t*_ is defined as follows:

1 $$\prod\nolimits_{{}} {PSPP\_START - W3_{t} = \, \max \left( {PSPP\_START_{t} , \, PSPP\_START_{t \, - \, 1} , \, PSPP\_START_{t \, - \, 2} } \right)}$$

On the other hand, the variable *CBPP3_START-W6*_*t*_ is defined as follows:

2 $$CBPP3\_START - W6_{t} = \, \max \left( {CBPP3\_START_{t} , \, CBPP3\_START_{t \, - \, 1} , \, CBPP3\_START_{t \, - \, 2} , \, CBPP3\_START_{t \, - \, 3} , \, CBPP3\_START_{t \, - \, 4} , \, CBPP3\_START_{t \, - \, 5} } \right)$$

To evaluate the impact of the unconventional monetary policy measures of the ECB as well as the four above-distinguished control variable categories on the daily rates of return on exchange rates, daily changes of 10-year sovereign bond yields and daily rates of return on stock market indexes, we proposed the estimation of the parameters of the following VARX–asymmetric generalized dynamic conditional correlation (AGDCC)-GARCH model (Cappiello et al. 2006)^2^:

3.a $$y_{t} = \sum\nolimits_{(p = 1)}^{P} {\prod\nolimits_{p} {y_{(t - p)} } } + \left[ {\begin{array}{*{20}c} \psi & \Lambda \\ \end{array} } \right] \, \left[ {\begin{array}{*{20}c} {x_{t} } \\ {m_{{EA,{\text{ t}}}} } \\ \end{array} } \right] + \varepsilon_{t} ,$$

with ${y}_{t}$ denoting a nine-dimensional vector of endogenous variables (daily rates of return on exchange rates, daily changes of 10-year sovereign bond yields and daily rates of return on stock market indexes for the three countries), while ${x}_{t}$ denotes a vector of monetary policy measures undertaken by the national banks of the Czech Republic, Hungary and Poland; macroeconomic surprises for some key macroeconomic indicators of the CEE-3 countries; and VSTOXX, daily rates of return on DAX and daily changes of 10-year German sovereign bond yields. ${m}_{EA,t}$ is the vector of variables associated with monetary policy measures launched by the ECB. Matrix $\Psi$ consists of parameters reflecting the impact of control variables on daily rates of return on exchange rates, daily changes of 10-year sovereign bond yields and daily rates of return on stock market indexes for the three countries. In turn, the effects of monetary policy measures launched by the ECB on stock, currency and bond markets in the CEE-3 countries are measured by parameters of the matrix $\Lambda$. We introduced an index for each market *i* = {*EXR*, *10Y*, *EQ*}, with *EXR* corresponding to the exchange rate market, *10Y* corresponding to the 10-year government bonds market and *EQ* corresponding to the equity market. Moreover, we introduced an index for each country *c* = *{PL, HU, CZ},* with *PL* corresponding to Poland, *HU* corresponding to Hungary and *CZ* corresponding to the Czech Republic. Since the vector ${y}_{t}$ consists of variables that defined changes for each market and each country, we introduced an index:

$$n = \, \left\{ {EXR\_PL, \, EXR\_HU, \, EXR\_CZ, \, 10Y\_PL, \, 10Y\_HU, \, 10Y\_CZ, \, EQ\_PL, \, EQ\_HU, \, EQ\_CZ} \right\}.$$

For example, ${y}_{t}^{EXR\_PL}$ defines the rate of return on exchange rate EUR/PLN at day *t*. In turn, ${y}_{t}^{EQ\_HU}$ defines the rate of return on BUX at day *t*.

In the case of the vector ${\varepsilon }_{t}$ from Eq. 3.a, it is assumed that:

3.b $$E(\varepsilon_{t} \varepsilon_{t}^{T} ) = H_{t} ,$$

where the covariance matrix is decomposed as follows:

3.c $$H_{t} = D_{t} R_{t} D_{t} ,$$

where the matrix ${D}_{t}$ consists of squared roots of variances of shocks:

3.d $$D_{t} = diag\left( {\begin{array}{*{20}c} {\sqrt {h_{EXR\_PL,t} } } & \ldots & {\sqrt {h_{EQ\_CZ,t} } } \\ \end{array} } \right)$$

These variances of shocks are modeled using the GJR-GARCH(1,1) model:

3.e $$h_{nn,t} = \alpha_{0n} + \alpha_{1n} \varepsilon_{n,t - 1}^{2} + \gamma_{1n} \varepsilon_{n,t - 1}^{2} I\left\{ {\varepsilon_{n,t - 1} < 0} \right\} + \beta_{1n} h_{nn,t - 1}$$

Correlations between shocks are time-varying and depend upon positive and negative shocks.

3.f $$R_{t} = (diag(Q_{t} ))^{( - 1/2)} Q_{t} (diag(Q_{t} ))^{( - 1/2)} ,$$

3.g $$Q_{t} = \left( {1 - \overline{\alpha }_{1} - \overline{\beta }_{1} } \right)\overline{Q} + \overline{\gamma }_{1} \left( {\overline{Q} - \overline{Q}^{ - } } \right) + \overline{\alpha }_{1} u_{t - 1} u_{t - 1}^{T} + \overline{\beta }_{1} Q_{t - 1} + \overline{\gamma }_{1} u_{t - 1}^{ - } \left( {u_{t - 1}^{ - } } \right)^{T}$$

The elements of vector ${u}_{t}$ were defined as follows:

3.h $$u_{n,t} = \frac{{\varepsilon_{nt} }}{{\sqrt {h_{nn,t} } }}$$

where ${u}_{t-1}^{-}$ consists of zero-threshold standardized errors and the matrices $\stackrel{-}{Q}$ and ${\stackrel{-}{Q}}^{-}$ are the unconditional covariance matrices of vectors ${u}_{t}$ and ${u}_{t}^{-}$, respectively.

The VARX-AGDCC-GARCH model seems to be an appropriate specification because of the fact that we used daily data. The sample period covers phases of higher and lower tensions related to the euro-area sovereign debt crisis. Moreover, shocks from different markets may be correlated, covariances among shocks may change over time and the impact of shocks may be asymmetric.

To evaluate the impact of the explanatory variables on uncertainty in the financial markets of the CEE-3 countries, volatilities in financial markets were calculated based on the estimation of the parameters of the following model:

4.a $$y_{t} - \overline{y} = \varepsilon_{t} ,$$

4.b $$E(\varepsilon_{t} \varepsilon_{t}^{T} ) = H_{t} ,$$

where decomposition of the matrix ${H}_{t}$ is given by Eqs. 3.c through 3.h.

After the estimation of the model (4.a and 4.b), the diagonal elements of matrix ${H}_{t}$ were extracted. Next, parameters of the following models explaining variances were estimated:

5 $$h_{nn,t} = \mu_{n} + B_{n} z_{t} + \xi_{n,t}$$

where ${h}_{nn,t}$ is the *n*-th diagonal element of the matrix ${H}_{t}$, ${z}_{t}$ collects additional explanatory variables associated with the ECB’s monetary policy measures and control variables, and ${B}_{n}$ is the matrix of parameters reflecting the impact of these variables on market volatilities.

Following the estimation of the parameters of the model (3.a–3.h) and estimation of the parameters of the model (5), linkages among different financial markets in different countries were studied. To do so, impulse response functions were constructed.

## Results and discussion

To find the optimal lag level of the VARX-AGDCC-GARCH model, Akaike, Bayesian Schwarz and Hannan–Quinn information criteria were used. Table 2 presents the values of the criteria for different lag levels.

**Table 2:** Selecting the optimal lag-length values of the information criteria

Lag length	Akaike information criteria	Hannan–Quinn information criteria	Bayesian Schwarz information criteria
1	− 51.525	− 51.268	− 51.166
2	− 51.474	− 51.260	− 51.057
3	− 51.402	− 51.231	− 50.926
4	− 51.382	− 51.194	− 50.787

Source: Authors’ own calculations

According to the results presented in Table 2, the optimal lag length equals 1. Therefore, the parameters of the model (3.a–3.h) were estimated for *p* = *1*.

In the model (3.a–3.h), it was assumed that there were spillovers occurring among the three countries and among the three markets. The presence of spillovers may be verified on the basis of imposing null restrictions on selected parameters of the matrix ${\Pi }_{1}$. After imposing null restrictions, they were verified with the use of the Wald test and the p-values turned out to be equal 0.000. This means that restrictions assuming a lack of spillovers among countries and among markets were not valid and the estimation of the parameters of the model for nine endogenous variables (three countries × three markets) was justified.

In the model (3.a–3.h), an asymmetric impact of shocks on volatilities and covariances was assumed. The use of parameters associated with asymmetry was justified when this asymmetry occurred. Therefore, the validity of the hypothesis ${\gamma }_{1n}=0$ was tested for all nine variants. Moreover, the validity of the hypothesis ${\stackrel{-}{\gamma }}_{1}=0$ was tested as well. In all 10 cases, the H0 hypothesis (assuming symmetric effects of shocks) was rejected with a p-value of less than 0.01. Therefore, parameters of the model assuming asymmetric effects of shocks were estimated.

Though spillovers among countries and among markets were taken into account and parameters of the model with nine-dimensional endogenous variables were estimated in this research, we herein present results individually for three countries. The design of a table that includes results for all countries, all markets and four variants of lag lengths would be too large. Tables 3 through 5 present estimates of parameters for variables associated with the ECB’s measures.^3^ In the case of insignificant variables (p > 0.1), we put ‘–’. It should be stressed that an explanatory variable was included in the tables if it was deemed significant for at least one market and one variant of lag-length, while variables insignificant for all variants and all lag lengths were not included in the tables.

**Table 3:** Estimates of parameters for variables associated with anticrisis measures of the ECB in equations explaining exchange rate returns, changes in sovereign bond yields and stock market returns for Poland

	Exchange rate returns				Changes in sovereign bond yields				Stock market returns			
Window Variable	1	3	6	11	1	3	6	11	1	3	6	11
*ABSPP_START*	–	− 0.003***	− 0.002*	–	− 0.052*	–	–	–	–	–	− 0.003*	–
*APP-END_ANNOUNCEMENT*	–	–	–	–	− 0.059**	− 0.035**	− 0.021**	− 0.016**	–	–	–	–
*APP-RESTART_ANNOUNCEMENT*	–	–	–	–	− 0.148***	–	–	–	–	–	–	–
*CBPP2_ANNOUNCEMENT*	–	–	–	–	− 0.102**	− 0.076***	− 0.061***	–	–	–	–	–
*CBPP3-ABSPP_ANNOUNCEMENT*	–	–	–	–	− 0.097**	− 0.068**	− 0.046**	− 0.042**	–	–	–	–
*CBPP3-ABSPP_DETAILS*	–	–	–	–	− 0.060*	–	–	–	–	–	–	–
*CSPP_ANNOUNCEMENT*	–	− 0.004**	–	–	–	–	–	–	–	–	–	–
*CSPP_START*	–	–	–	–	− 0.044*	–	–	–	–	–	–	–
*LTRO_ANNOUNCEMENT*	0.011**	–	–	–	–	–	–	–	–	–	–	–
*LTRO(1)_ALLOTMENT*	0.005***	0.005**	–	–	− 0.059**	–	–	–	0.015***	0.005*	–	–
*OMT_ANNOUNCEMENT*	− 0.011**	− 0.005*	− 0.002*	− 0.002*	− 0.049*	− 0.038*	− 0.029**	–	–	–	–	–
*OMT_DETAILS*	− 0.019***	− 0.005**	− 0.003**	− 0.002*	–	–	–	–	–	–	–	–
*PSPP_ANNOUNCEMENT*	− 0.011***	− 0.004*	–	–	–	–	–	–	–	–	–	–
*SMP_ANNOUNCEMENT*	− 0.023*	–	–	–	–	–	–	–	0.023***	–	–	–
*TLTRO(1)_ANNOUNCEMENT*	–	–	–	–	–	− 0.042*	–	–	–	–	–	–
*TLTRO(2)_ANNOUNCEMENT*	–	–	–	–	–	–	–	–	0.013**	–	–	–

Source: Authors’ own calculations

^*^, ** and *** indicate statistical significance at the 0.1, 0.05 and 0.01 levels, respectively. The symbol ‘−’ denotes statistical insignificance

The results of the estimation of the parameters of the model (3.a–3.h) for exchange rate markets of the CEE-3 countries indicate that these markets have experienced a strong degree of influence from the ECB’s asset purchase programs, including the sovereign bonds of the euro-area countries (Tables 3, 4 and 5). The announcements of the SMP, OMT and PSPP resulted in the appreciation of the local currencies vis-à-vis the euro (the only exception being the insignificance of PSPP for the Czech Republic). The obtained results were largely consistent with the findings of prior empirical works (Falagiarda et al. 2015; Ciarlone and Colabella 2016; Fratzscher et al. 2016). Notably, the strongest effects were identified for the SMP. At the same time, the OMT was proven to be the most durable. The *OMT_ANNOUNCEMENT* and *OMT_DETAILS* variables turned out to be significant also in longer windows (6 or 11 days), when the effects of the SMP and PSPP largely vanish.

**Table 4:** Estimates of parameters for variables associated with the anticrisis measures of the ECB in equations ex–laining exchange rate returns, changes in sovereign bond yields and stock market returns for Hungary

	Exchange rate returns				Changes in sovereign bond yields				Stock market returns			
Window Variable	1	3	6	11	1	3	6	11	1	3	6	11
*ABSPP_START*	–	–	–	–	–	–	–	–	–	–	− 0.007**	–
*APP-RESTART_ANNOUNCEMENT*	–	–	–	–	− 0.139**	–	–	–	–	–	–	–
*CBPP2_ANNOUNCEMENT*	–	–	–	–	− 0.191**	− 0.141**	− 0.088*	− 0.051*	–	–	–	–
*CBPP3_START*	–	–	–	–	–	–	–	–	− 0.013**	− 0.010***	− 0.007**	− 0.004*
*LTRO_ANNOUNCEMENT*	0.018**	–	–	–	–	–	–	–	–	–	–	–
*LTRO(1)_ALLOTMENT*	0.010**	0.008**	0.006**	0.005**	–	–	–	–	0.004***	–	–	–
*LTRO(2)_ALLOTMENT*	–	–	–	–	− 0.164*	–	–	–	–	–	–	–
*OMT_ANNOUNCEMENT*	− 0.016***	− 0.012***	− 0.004*	− 0.004*	− 0.110***	− 0.141**	–	–	–	–	–	–
*PSPP_ANNOUNCEMENT*	− 0.009**	–	–	–	–	–	–	–	0.011***	–	–	–
*SMP_ANNOUNCEMENT*	− 0.026*	–	–	–	–	–	–	–	0.083***	–	–	–
*TLTRO(1)_ANNOUNCEMENT*	− 0.007***	–	–	–	–	–	–	–	–	–	–	–

Source: Authors’ own calculations

^*^, ** and *** indicate statistical significance at the 0.1, 0.05 and 0.01 levels, respectively. The symbol ‘–’ denotes statistical insignificance

**Table 5:** Estimates of parameters for variables associated with anticrisis measures of the ECB in equations explaining exchange rate returns, changes in sovereign bond yields and stock market for the Czech Republic

	Exchange rate returns				Changes in sovereign bond yields				Stock market returns			
Window Variable	1	3	6	11	1	3	6	11	1	3	6	11
*ABSPP_START*	− 0.003***	-	-	–	–	–	–	− 0.010*	–	–	− 0.005**	–
*APP-END_ANNOUNCEMENT*	–	–	–	–	–	–	–	–	–	–	− 0.005*	− 0.002*
*APP-EXTENSION_ANNOUNCEMENT*	–	–	–	− 0.002*	–	–	–	–	–	–	–	–
*CBPP2_ANNOUNCEMENT*	–	–	–	–	–	–	–	− 0.036**	0.028**	–	–	–
*CBPP3-ABSPP_ANNOUNCEMENT*	–	–	–	− 0.001*	–	–	–	–	–	–	–	–
*CBPP3-ABSPP_DETAILS*	–	–	–	–	− 0.029*	–	–	–	–	–	–	–
*CBPP3_START*	–	–	0.001**	0.001**	–	–	–	–	–	–	0.005*	–
*CSPP_START*	–	–	–	–	–	–	–	–	− 0.024***	− 0.017***	− 0.011***	–
*LTRO(1)_ALLOTMENT*	0.003***	–	–	–	–	–	–	–	0.017***	0.011***	0.007***	0.004**
*LTRO(2)_ALLOTMENT*	0.002***	–	–	–	–	–	–	–	0.019***	0.008*	–	–
*OMT_ANNOUNCEMENT*	–	–	–	–	− 0.110***	− 0.059**	− 0.033**	–	–	–	–	–
*OMT_DETAILS*	− 0.005*	− 0.004**	− 0.003**	− 0.002**	–	–	–	–	–	–	–	–
*SMP_ANNOUNCEMENT*	–	–	− 0.005**	–	–	–	–	–	0.054***	–	–	–
*TLTRO(2)_ANNOUNCEMENT*	–	–	–	–	–	–	–	–	0.018***	–	–	–
*TLTRO(3)_ANNOUNCEMENT*	–	–	–	–	–	–	–	–	0.008*	0.005**	–	–

Source: Authors’ own calculations

*, ** and *** indicate statistical significance at the 0.1, 0.05 and 0.01 levels, respectively. The symbol ‘–’ denotes statistical insignificance

When it comes to the ECB’s other asset purchase programs, we found the spillover effects of the ABSPP led to the appreciation of PLN and CZK against the euro (though the moment of their occurrence and durability differed). The impact of the announcements related to the two editions of the CBPP, APP and CSPP turned out to be negligible and heterogeneous across the analyzed countries. Referring to the nonstandard ECB policies aimed at offering banks long-term funding with attractive conditions, our results suggest that all the CEE-3 currencies experienced downward pressure of 3Y LTROs. At the same time, we identified cross-country heterogeneities concerning the TLTRO. In particular, the announcements of all three TLTRO series resulted in the appreciation of HUF vis-à-vis the euro, while, for Poland and the Czech Republic, these operations played only a marginal or no role at all. Finally, somewhat surprisingly, we essentially observed no significant spillovers from the ECB’s conventional monetary policy measures to the exchange rate markets of the CEE-3 countries.

Turning to the spillovers from the ECB’s monetary policies to sovereign bond markets of the CEE-3 countries, our results suggest that these have been less significant in comparison with the effects identified for the exchange rate markets. We also noted greater heterogeneities across the CEE-3 countries with respect to their reactions to particular initiatives. At the same time, however, the vast majority of ECB measures that turned out to be significant resulted in lower yields. The latter result may indicate a positive correlation between confidence in the euro area (improved by the ECB’s policies) and that in the analyzed countries. Our results may support the conclusion stemming from the study of Korus (2019), which was conducted among a set of Nordic countries, that the international transmission of the ECB’s monetary policies associated with sovereign-bond purchase programs operated via the confidence channel.

Of the nonstandard ECB’s measures involving the purchasing of sovereign securities, the OMT announcement played an important role in reducing Polish, Hungarian and Czech sovereign bond yields. The spillovers from the SMP and PSPP announcements turned out to be more limited—although, in the case of the latter, the significance of the APP restart variable for Poland and Hungary is noteworthy. When it comes to other asset purchase programs, the yields of all CEE-3 countries have been sensitive to the announcements related to CBPP3 and ABSPP, particularly when considering Poland. Finally, measures aimed at supporting bank lending in the euro area showed limited impact as only some variables related to 3Y LTROs proved statistically significant. Conventional interest-rate changes seem not to have shaped the yields under consideration.

Upon assessing the durability of the ECB’s spillovers, such seems to have been low. Most of the announcements, which were found to be important determinants of the CEE-3 sovereign bond yields, exerted influence only in variants of one-day or three-day windows.

Focusing on the reactions of stock market indices, we found that these have increased in all the CEE-3 countries because of the announcement of the SMP. The relevant spillovers turned out not to be durable, however, as they vanished within a three-day window. Moreover, we identified similar effects in the case of the PSPP announcement for Hungary. Interestingly, we observed no statistically significant coefficient for the events related to the OMT, which seemed to have had the most pronounced spillovers for exchange rates and long-term sovereign bond yields. Similar to the other two financial instruments, the sensitivity of equity prices to the ECB’s other asset purchase programs varied across the countries considered and with regard to the direction of impact; the only exception was the negative impact of the start of the ABSPP program. Turning to the nonstandard liquidity-providing operations, we can conclude that both 3Y LTRO and TLTRO have generally raised stock indices. Hence, our results are only partially in line with those of Fratzscher et al. (2016), who identified positive gains in bank equity but no positive impact of 3Y LTRO on broad equity indices for the emerging European Union. In the period under analysis, the stock markets of CEE-3 countries remained largely unaffected by the ECB’s conventional monetary policy measures.

Tables 6, 7 and 8 present information concerning the significance of variables associated with anticrisis measures as well as the conventional monetary policies of the ECB in equations explaining volatilities in currency, bond and stock markets.

**Table 6:** Estimates of parameters for variables associated with the ECB measures in equations explaining volatilities in the currency market, bond market and stock market of Poland

	Currency market				Bond market				Stock market			
Window Variable	1	3	6	11	1	3	6	11	1	3	6	11
*ABSPP_START*	− 0.001***	− 0.001***	− 0.001*	–	− 0.009*	–	–	–	− 0.003***	− 0.003***	− 0.003***	− 0.003***
*APP-END_ANNOUNCEMENT*	–	–	–	–	0.014**	–	–	–	0.004***	0.004***	0.004***	0.002***
*APP-EXTENSION_ANNOUNCEMENT*	–	–	–	–	–	0.014**	0.014***	0.01***	–	0.001*	0.001**	0.001*
*APP-RESTART_ANNOUNCEMENT*	0.001***	0.002***	0.001***	0.001***	0.035***	0.044***	0.043***	0.030***	0.007***	0.007***	0.007***	0.005***
*CBPP2_ANNOUNCEMENT*	–	–	–	–	− 0.021***	− 0.015***	− 0.010**	− 0.011**	–	–	–	–
*CBPP2_DETAILS*	− 0.001***	− 0.001***	− 0.001**	− 0.001**	− 0.012***	− 0.015***	− 0.039***	− 0.044***	− 0.003***	− 0.004***	− 0.005***	− 0.007***
*CBPP3-ABSPP_ANNOUNCEMENT*	0.001***	0.001***	0.001***	–	0.010***	0.013***	0.019***	0.028***	0.004***	0.005***	0.005***	0.003***
*CBPP3_START*	− 0.003***	− 0.002***	− 0.002***	− 0.002***	− 0.023***	− 0.023***	–	–	− 0.007***	− 0.005***	− 0.004***	− 0.004***
*CSPP_ANNOUNCEMENT*	− 0.003***	− 0.002**	− 0.001***	− 0.001*	− 0.017***	− 0.028***	− 0.028***	− 0.029***	− 0.008***	− 0.003***	− 0.003***	− 0.003***
*CSPP_DETAILS*	− 0.001***	− 0.001*	–	–	− 0.012***	− 0.011***	− 0.009***	− 0.011***	− 0.001***	− 0.002***	− 0.003***	− 0.005***
*CSPP_START*	− 0.002***	− 0.003***	− 0.005***	− 0.005***	− 0.016***	− 0.019***	− 0.027***	− 0.018***	− 0.002***	− 0.004***	− 0.009***	− 0.011***
*INT-RATE-DOWN_ECB*	–	–	–	–	–	–	–	–	–	− 0.001**	− 0.001**	–
*INT-RATE-UP_ECB*	–	–	–	–	− 0.014***	− 0.013***	− 0.012***	− 0.013***	–	− 0.004***	− 0.005***	− 0.005***
*LTRO_ANNOUNCEMENT*	− 0.005***	− 0.004***	− 0.004***	− 0.002***	–	–	–	− 0.012***	− 0.007***	− 0.004***	− 0.003***	− 0.003***
*LTRO(1)_ALLOTMENT*	− 0.002***	− 0.002***	− 0.002***	− 0.003***	− 0.014***	− 0.015***	− 0.019***	− 0.027***	− 0.002***	− 0.002***	− 0.003***	− 0.004***
*LTRO(2)_ALLOTMENT*	− 0.001***	− 0.001*	− 0.001*	–	− 0.023***	− 0.020***	− 0.024***	− 0.020***	− 0.005***	− 0.005***	− 0.007***	− 0.005***
*OMT_ANNOUNCEMENT*	− 0.003***	− 0.002***	− 0.001**	–	− 0.008***	–	–	–	− 0.003***	− 0.003***	− 0.004***	− 0.004***
*OMT_DETAILS*	− 0.002***	− 0.002***	− 0.001*	− 0.001*	− 0.012***	− 0.013***	− 0.015***	− 0.013***	− 0.005***	− 0.004***	− 0.004***	− 0.005***
*PSPP_ANNOUNCEMENT*	− 0.002***	− 0.002**	− 0.001**	− 0.001**	− 0.011***	–	–	–	− 0.001***	− 0.001**	− 0.001***	− 0.003***
*PSPP_START*	− 0.001***	–	–	–	–	–	–	–	–	–	− 0.002***	–
*SMP_ANNOUNCEMENT*	− 0.001*	− 0.001*	–	–	− 0.023***	–	–	–	− 0.010***	− 0.004***	− 0.004*	− 0.007***
*TLTRO(1)_ANNOUNCEMENT*	0.001***	0.002***	0.002***	0.003***	0.010***	0.010***	0.017***	0.012***	0.002***	0.004***	0.004***	0.002***
*TLTRO(3)_ANNOUNCEMENT*	0.001***	0.001***	0.001***	0.002***	0.003***	–	–	–	0.003***	0.003***	0.003***	0.003***

Source: Authors’ own calculations

*, ** and *** indicate statistical significance at the 0.1, 0.05 and 0.01 levels, respectively. The symbol ‘–’ denotes statistical insignificance

**Table 7:** Estimates of parameters for variables associated with the ECB measures in equations explaining volatilities in the currency market, bond market and stock market of Hungary

	Currency market				Bond market				Stock market			
Window Variable	1	3	6	11	1	3	6	11	1	3	6	11
*ABSPP_START*	− 0.001***	− 0.001***	− 0.001***	− 0.001***	− 0.001**	− 0.001***	–	–	–	–	–	–
*APP-END_ANNOUNCEMENT*	− 0.001***	− 0.001***	− 0.001***	− 0.002***	− 0.003***	− 0.003***	− 0.004***	− 0.008***	0.001*	–	–	–
*APP-EXTENSION_ANNOUNCEMENT*	–	− 0.001**	− 0.001***	− 0.001***	–	–	− 0.004*	− 0.005***	–	–	–	–
*APP-RESTART_ANNOUNCEMENT*	–	–	–	–	0.010***	0.014***	0.009***	0.005***	0.008***	0.010***	0.011***	0.010***
*CBPP2_ANNOUNCEMENT*	− 0.002***	− 0.002***	− 0.001***	− 0.001*	− 0.012***	− 0.010***	− 0.010***	− 0.017***	–	–	–	–
*CBPP2_DETAILS*	–	− 0.002***	− 0.001***	–	− 0.031***	− 0.029***	− 0.034***	− 0.034***	− 0.019***	− 0.021***	− 0.021***	− 0.018***
*CBPP3-ABSPP_ANNOUNCEMENT*	0.001***	–	–	–	0.008***	0.011***	0.008***	0.008***	0.009***	0.012***	0.012***	0.010***
*CBPP3-ABSPP_DETAILS*	–	–	–	–	− 0.004***	− 0.002***	− 0.002***	− 0.004***	–	–	–	–
*CBPP3_START*	− 0.003***	− 0.002***	− 0.001***	− 0.002***	–	–	–	–	− 0.012***	− 0.007***	− 0.005***	− 0.004***
*CSPP_ANNOUNCEMENT*	− 0.004***	− 0.002**	− 0.001**	− 0.001**	− 0.021***	− 0.011***	− 0.008***	− 0.007***	− 0.015***	− 0.006**	− 0.004**	–
*CSPP_DETAILS*	− 0.001***	− 0.002***	− 0.001***	− 0.002***	− 0.007***	− 0.008***	− 0.007***	− 0.008***	− 0.003***	− 0.006***	− 0.005***	− 0.008***
*CSPP_START*	− 0.002***	− 0.003***	− 0.005***	− 0.006***	− 0.014***	− 0.019***	− 0.028***	− 0.032***	− 0.009***	− 0.004***	− 0.024***	− 0.026***
*INT-RATE-DOWN_ECB*	–	–	–		–	− 0.003**	–	–	–	–	− 0.004***	− 0.004***
*INT-RATE-UP_ECB*	–	− 0.001***	− 0.001***	− 0.001***	− 0.004***	− 0.006***	− 0.009***	− 0.010***	–	–	− 0.005**	− 0.004*
*LTRO_ANNOUNCEMENT*	− 0.002***	− 0.002***	− 0.001***	− 0.001***	–	–	–	–	− 0.016***	− 0.009***	− 0.007***	− 0.010**
*LTRO(1)_ALLOTMENT*	− 0.003***	− 0.002***	− 0.001***	− 0.001***	–	–	–	–	− 0.007***	− 0.012**	− 0.017***	− 0.024***
*LTRO(2)_ALLOTMENT*	− 0.002***	− 0.001***	− 0.001***	− 0.001***	–	–	–	–	− 0.005***	− 0.004***	− 0.009***	− 0.007***
*OMT_ANNOUNCEMENT*	− 0.001*	− 0.001**	− 0.001*	− 0.001***	− 0.008***	–	–	–	− 0.011***	− 0.010***	− 0.011***	− 0.007***
*OMT_DETAILS*	− 0.001***	− 0.001**	− 0.001*	− 0.001***	− 0.008***	− 0.007***	− 0.008***	− 0.004***	− 0.010***	− 0.009***	− 0.010***	− 0.003*
*PSPP_ANNOUNCEMENT*	–	–	–	–	− 0.008***	− 0.007***	–	–	− 0.003***	–	–	–
*SMP_ANNOUNCEMENT*	− 0.002***	–	–	–	− 0.010***	–	–	–	–	–	–	–
*TLTRO(1)_ANNOUNCEMENT*	–	0.001***	0.001***	0.001***	0.014***	0.019***	0.017***	0.016***	0.007***	0.012***	0.013***	0.013***
*TLTRO(3)_ANNOUNCEMENT*	0.001***	0.001***	0.001**	0.001**	0.030***	0.026***	0.023***	0.019***	0.008***	0.008***	0.009***	0.009***

Source: Authors’ own calculations

*, ** and *** indicate statistical significance at the 0.1, 0.05 and 0.01 levels, respectively. The symbol ‘–’ denotes statistical insignificance

**Table 8:** Estimates of parameters for variables associated with the ECB measures in equations explaining volatilities in the currency market, bond market and stock market of the Czech Republic

	Currency market				Bond market				Stock market			
Window variable	1	3	6	11	1	3	6	11	1	3	6	11
*ABSPP_START*	–	–	–	–	− 0.007***	− 0.007***	− 0.006***	− 0.005***	0.003***	0.003***	0.003***	0.004***
*APP-END_ANNOUNCEMENT*	− 0.001***	− 0.001***	− 0.001***	− 0.001***	− 0.009***	− 0.009***	− 0.010***	− 0.013***	0.003***	0.002***	–	–
*APP-RESTART_ANNOUNCEMENT*	0.001*	–	–	–	0.031***	0.031***	0.027***	0.016***	0.012***	0.012***	0.012***	0.011***
*CBPP2_ANNOUNCEMENT*	–	− 0.002***	− 0.002***	− 0.002***	− 0.040***	− 0.037***	− 0.035***	− 0.038***	− 0.020***	− 0.010***	− 0.008***	− 0.016***
*CBPP2_DETAILS*	− 0.001***	− 0.005***	− 0.005***	− 0.004***	− 0.031***	− 0.030***	− 0.030***	–	− 0.030***	− 0.034***	− 0.037***	− 0.040***
*CBPP3-ABSPP_ANNOUNCEMENT*	0.001***	0.001**	–	–	0.012***	0.012***	0.011***	0.012***	0.009***	0.010***	0.010***	0.009***
*CBPP3_START*	− 0.001***	− 0.001***	–	–	− 0.013***	− 0.008***	− 0.032***	− 0.010***	− 0.003***	− 0.002***	–	–
*CSPP_ANNOUNCEMENT*	–	− 0.003**	− 0.003**	− 0.003***	− 0.015***	− 0.011***	− 0.008***	− 0.013***	− 0.015***	− 0.007**	− 0.005***	− 0.004***
*CSPP_DETAILS*	–	− 0.001***	− 0.001***	− 0.001***	− 0.010***	− 0.009***	− 0.011***	− 0.014***	− 0.001*	− 0.002**	− 0.003***	− 0.004***
*CSPP_START*	− 0.001***	− 0.002***	− 0.002***	− 0.003***	− 0.018***	− 0.023***	− 0.032***	− 0.035***	− 0.012***	− 0.016***	− 0.022***	− 0.025***
*INT-RATE-DOWN_ECB*	–	–	–	–	− 0.006*	− 0.007***	− 0.006***	–	–	–	–	–
*INT-RATE-UP_ECB*	–	–	–	–	− 0.009***	− 0.011***	− 0.013***	− 0.014***	− 0.003***	–	–	–
*LTRO_ANNOUNCEMENT*	− 0.001*	− 0.003**	− 0.003**	− 0.002**	–	–	–	–	− 0.035***	− 0.030***	− 0.027***	− 0.022***
*LTRO(1)_ALLOTMENT*	− 0.001***	− 0.001***	–	–	–	–	–	–	− 0.020***	− 0.017***	− 0.019***	− 0.019***
*LTRO(2)_ALLOTMENT*	− 0.001***	− 0.001***	–	–	− 0.010***	− 0.009***	− 0.013***	− 0.009***	− 0.002***	− 0.002***	− 0.004*	− 0.004**
*OMT_ANNOUNCEMENT*	− 0.001***	− 0.001***	− 0.001***	− 0.001***	–	–	–	–	− 0.011***	− 0.012***	− 0.014***	− 0.010***
*OMT_DETAILS*	− 0.001***	− 0.001***	–	–	− 0.007***	− 0.004***	− 0.003***	− 0.003***	− 0.008***	− 0.007***	− 0.007***	− 0.003***
*PSPP_ANNOUNCEMENT*	− 0.002***	− 0.001***	–	–	− 0.011***	–	–	–	− 0.007***	− 0.006***	− 0.006***	− 0.006***
*PSPP_START*	− 0.002***	− 0.002***	− 0.001**	–	–	− 0.006***	− 0.005***	–	–	–	–	–
*SMP_ANNOUNCEMENT*	–	–	–	–	− 0.020***	− 0.031***	− 0.043***	− 0.043***	− 0.021***	–	–	–
*TLTRO(1)_ANNOUNCEMENT*	0.001***	–	–	–	0.011***	0.011***	0.012***	0.006***	0.011***	0.014***	0.014***	0.012***
*TLTRO(3)_ANNOUNCEMENT*	–	–	–	–	0.005***	0.004**	0.004***	0.004***	0.013***	0.013***	0.014***	0.013***

Source: Authors’ own calculations

*, ** and *** indicate statistical significance at the 0.1, 0.05 and 0.01 levels, respectively. The symbol ‘–’ denotes statistical insignificance

The results presented in Tables 6 through 8 suggest that the announcements concerning most of the ECB’s initiatives resulted in a drop in uncertainty among the financial markets of the CEE-3 countries. This conclusion particularly applies to those measures involving purchases of euro-area sovereign bonds. Announcements related to the OMT (i.e., variables *OMT_ANNOUNCEMENT *and *OMT_DETAILS*) triggered a significant decrease in the uncertainty present in currency and stock markets in all analyzed countries. This finding confirms that not only did the OMT play a paramount role in resolving the euro-area sovereign debt crisis (Afonso and Kazemi 2018; Afonso and Jalles 2019) but it may also have positively influenced the financial markets in Central and Eastern Europe through the confidence channel.

The level of uncertainty in the financial markets of the CEE-3 countries also has been subjected to a positive influence of the PSPP; notably, this program exerted a stronger impact on currency and stock markets than on sovereign bond markets during the study sample period. The effects of the SMP turned out to be significant mainly in the case of the Polish markets.

Turning to the other ECB initiatives involving asset purchases, the CSPP announcement significantly reduced the level of volatility in the financial markets of the CEE-3 countries. Information concerning the start of the ABSPP program resulted in a decrease of volatility in sovereign bond markets in both the Czech Republic and Hungary as well as in the stock and currency markets in Poland. Announcements concerning CBPP2 and CBPP3, in turn, exerted ambiguous effects on financial markets’ uncertainty in the countries under consideration. LTROs affected the volatility of financial markets in the CEE-3 countries as well. When it comes to the conventional ECB measures, increases in the interest rate turned out to be significant in more variants than interest rate decreases.

Following the estimation of the parameters of the VARX-AGDCC-GARCH model, impulse response functions were calculated and are presented in Appendix 2.

## Conclusions

In this paper, the spillover effects from the (un)conventional monetary policy announcements of the ECB to the exchange rate, sovereign bond and stock markets of the Czech Republic, Hungary and Poland were investigated. Specifically, the parameters of the VARX-AGDCC-GARCH model were estimated. Moreover, variances reflecting uncertainty in financial markets were extracted and later modeled.

The results of the estimation revealed that the financial markets of the CEE-3 countries have been subjected to a strong influence of the ECB’s monetary policy announcements. This finding particularly pertains to the ECB’s initiatives involving purchases of euro-area sovereign debt. For this category of measures, highly similar effects were identified for all analyzed countries. For the currency markets, the announcements related to the SMP, OMT and PSPP led the local currencies to appreciate against the euro. In the case of the sovereign bond markets, the announcement of the OMT resulted in decreasing yields and reductions in uncertainty across the CEE-3 countries. In other words, it seems that the program directly aimed at reducing the sovereign bond yields of the most vulnerable EMU countries at the height of the crisis also had positive ‘side effects’ for some non–euro-area E.U. members. The relatively limited spillovers from the PSPP may be explained in the context of already lower levels of uncertainty and interest rates in the EMU at the moment of its announcement and introduction. Regarding the stock markets, the strongest effects were linked to the SMP as all the analyzed indices recorded increases following its announcement. These effects, however, have not been proven to be durable.

Furthermore, similarities across the CEE-3 countries have been observed with regard to the sensitivity of their financial markets to the ECB’s conventional monetary policy measures; specifically, all of them turned out to be unaffected by the ECB’s interest rate changes. Finally, focusing on the spillover effects from other nonstandard ECB policies, a much more heterogeneous impact has been identified regarding their (non)significance, moment of occurrence and durability.

Taking the above into consideration, it seems that the financial markets of the CEE-3 countries have responded primarily to the most pronounced and innovative ECB measures, which were often introduced amidst the highest tensions in the euro-area sovereign debt crisis and whose launch was accompanied by numerous controversies. Regarding the ‘newest’ initiatives, which have not been accounted for in previous studies, the ECB’s decision to end the APP has played a discernible role in affecting the financial markets of CEE-3 countries.

The presented results concerning the impact of the ECB’s monetary policy measures on the currency markets of the CEE-3 countries are strongly in line with conclusions from previous research analyses. In turn, where sovereign bond and stock markets are concerned, we can confirm some yet contradict other existing findings. These differences might result from various lengths of the research samples as well as the fact that we also accounted for linkages among the markets and the CEE-3 countries, which previous studies did not attempt to do. In general, in economic literature, findings concerning the impact of ECB initiatives on these markets in Central and Eastern Europe are not conclusive, necessitating more extensive analyses concerning this issue to be conducted in the future.

Finally, as both this study and previous works have identified cross-country heterogeneities, further analyses could place a greater emphasis on the sources of these discrepancies. In particular, the CEE-3 countries are similar to one another in their level of economic development, monetary policy framework and exchange rate arrangements. However, a closer look at their macro-financial ties with the euro-area countries could shed some additional light.

## Figures and Tables

**Fig. 1:**
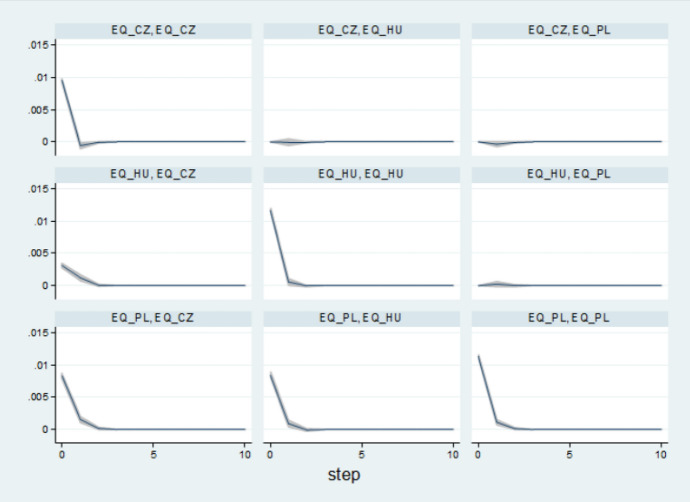
Impulse-response analysis between countries (equity markets). Source: Authors’ own calculations.

**Fig. 2:**
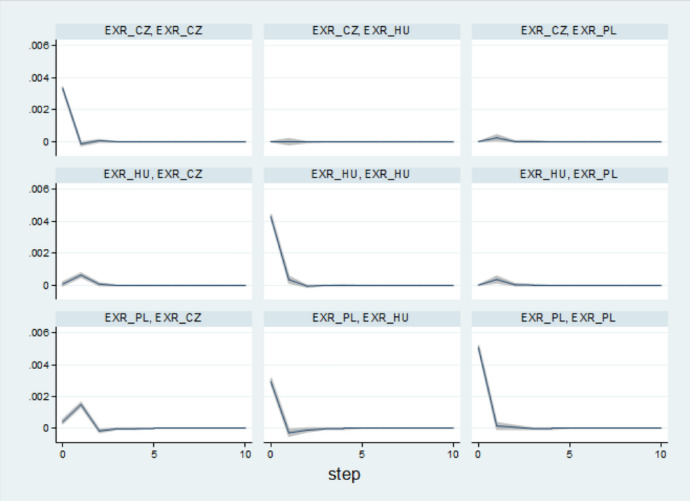
Impulse-response analysis between countries (currency markets). Source: Authors’ own calculations.

**Fig. 3:**
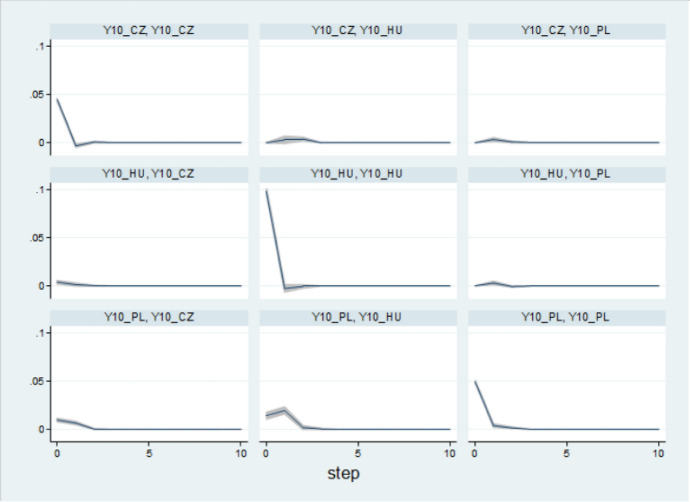
Impulse-response analysis between countries (bond markets). Source: Authors’ own calculations.

**Fig. 4:**
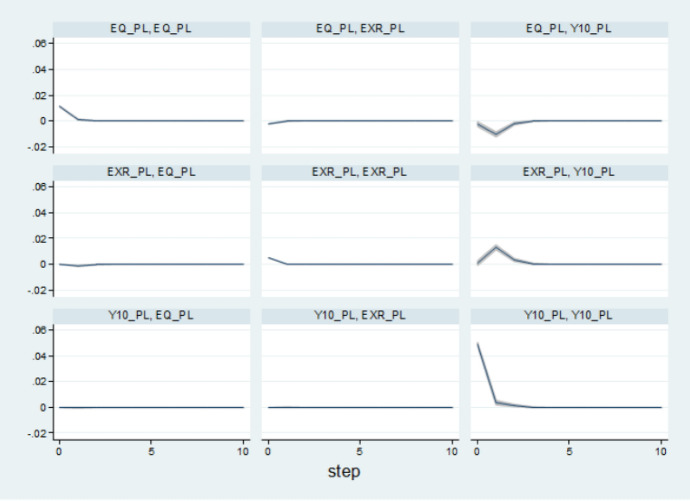
Impulse-response analysis between markets (Poland). Source: Authors’ own calculations.

**Fig. 5:**
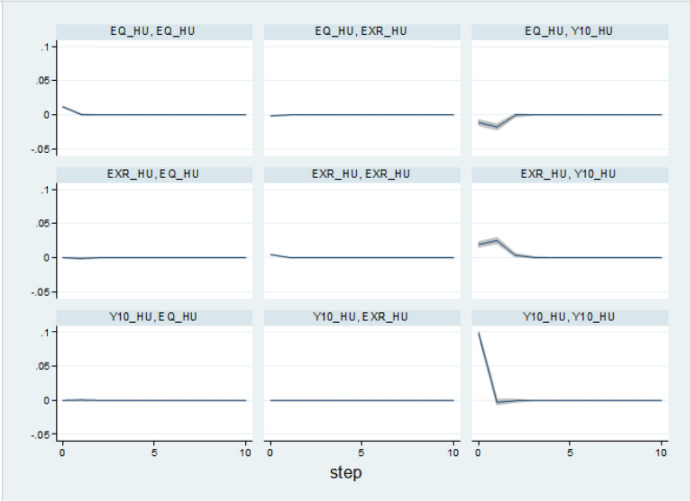
Impulse-response analysis between markets (Hungary). Source: Authors’ own calculations.

**Fig. 6:**
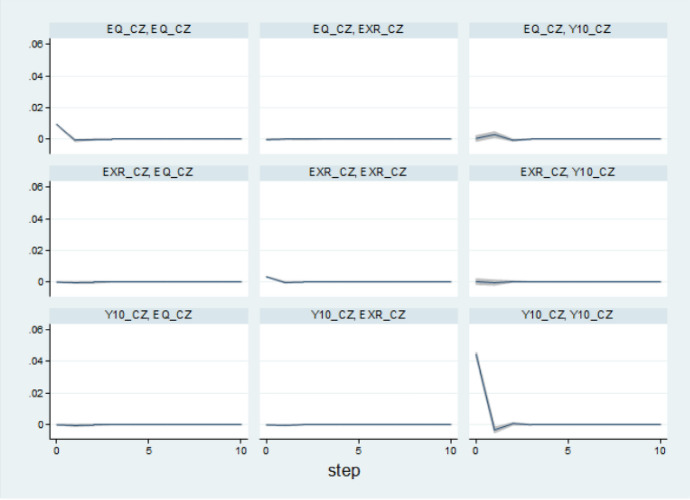
Impulse-response analysis between markets (the Czech Republic). Source: Authors’ own calculations.

## Appendix 1

Apart from variables associated with unconventional measures of the European Central Bank (ECB), control variables associated with domestic monetary policy measures in the CEE-3 countries, the performance of the financial markets in Germany, global risk-aversion and macroeconomic surprises are used as explanatory ones. Herein, the construction of these variables is described.

### Domestic monetary policy measures in the CEE-3 countries

It is worth noting that nonstandard monetary policy measures were also conducted by the national banks of the CEE-3 countries. The vast majority of them were launched by the Magyar Nemzeti Bank (MNB) and are as follows:

The funding for growth scheme (FGS), announced in April 2013, was aimed at supporting the SME sector in accessing forint-denominated loans and strengthening financial stability.

The mortgage bond purchase program (MBPP), announced in November 2017, was aimed at triggering an increase in mortgage bond issues and boosting market activity.

The self-financing program (SFP), launched in April 2014, was aimed at reducing gross external debt by moving toward forint financing of the government.

The corporate bond purchasing program (CBPP), initiated in July 2019, aimed at supporting the diversification of the funding to the domestic corporate sector.

When it comes to the unconventional monetary policy measures of the Czech National Bank (CNB), we considered the CNB’s commitment of November 2013 to intervene in the foreign exchange market so as to keep the exchange rate close to CZK 27/EUR. Interventions in the foreign exchange market were also performed by the National Bank of Poland (NBP). These were, however, of a more occasional nature. In particular, the NBP purchased foreign currency in April 2010 and conducted several interventions selling foreign currencies in exchange for zlotys in 2011.

Like in the case of the ECB, we also included the interest rate changes introduced by the national banks of the CEE-3 countries. Table 9 presents the names and definitions of the variables associated with the domestic monetary policies of the CNB, MNB and NBP.

**Table 9:** Variables associated with CZK, MNB and NBP measures

Measure	Date	Event	Variable
Interest rate policy	68 decisions on key interest rates including 4 (8), 36 (5) and 10 (5) downgrades (upgrades) for the Czech Republic, Hungary and Poland, respectively	NBP decides to lower its key interest rates	*PL_INTRATE-DOWN*
		CNB decides to lower its key interest rates	*CZ_INTRATE-DOWN*
		MNB decides to lower its key interest rates	*HU_INTRATE-DOWN*
		NBP decides to increase its key interest rates	*PL_INTRATE-UP*
		CNB decides to increase its key interest rates	*CZ_INTRATE-UP*
		MNB decides to increase its key interest rates	*HU_INTRATE-UP*
FGS	4 April 2013	MNB announces FGS	*FGS_ANNOUNCEMENT*
	1 June 2013	MNB launches its three-pillar FGS	*FGS_START*
	31 March 2017	FGS comes to an end	*FGS_END*
MBPP	21 November 2017	MNB announces MBPP	*MBPP_ANNOUNCEMENT*
	21 December 2017	Terms and conditions of purchases are announced	*MBPP_DETAILS*
	18 September 2018	MNB decides to phase out MBPP by the end of 2018	*MBPP-END_ ANNOUNCEMENT*
SFP	23 April 2014	MNB announces SFP	*SFP_ANNOUNCEMENT*
	10 May 2016	MNB announces termination of IRS tenders as of 7 July 2016	*SFP-END_ANNOUNCEMENT*
	7 July 2016	The date of the last IRS tender	*SFP_END*
CBPP	26 March 2019	MNB announces CBPP	*HUCBPP_ANNOUNCEMENT*
	1 July 2019	CBPP is launched	*HUCBPP_START*
Exchange rate commitment of CNB	7 November 2013–6 April 2017	The period of exchange rate floor of CZK27 = EUR1	*ERC*
NBP FX interventions	9 April 2010	NBP purchases a certain amount of foreign currency	*PL-INTERVENTION_PUR*
	23 September 2011, 30 September 2011, 3 October 2011, 23 November 2011, 29 December 2011	NBP sells a certain amount of foreign currency	*PL-INTERVENTION_SEL*

Source: Authors’ own compilation based on CZK, MNB and NBP press releases.

### Daily rates of return on DAX, daily changes in 10-year German sovereign bond yields and VSTOXX

The performance of the stock markets in the CEE-3 countries is likely to largely depend on the performance of the stock market in Germany. Therefore, the daily rates of return in DAX are included as an explanatory control variable. Similarly, the performance of sovereign bond markets in the countries under analysis should depend on the performance of the sovereign bond market in Germany. Based on this reason, daily changes of 10-year German sovereign bond yields were also incorporated into the set of explanatory variables. Moreover, the volatility of the European equity markets could also affect changes in the prices of financial instruments as well as their volatilities in the CEE-3 countries, so the VSTOXX index was also considered as a control variable.^4^

### Macroeconomic surprises

To minimize the risk of the occurrence of the omitted variable bias problem, we also included macroeconomic surprises in our set of explanatory variables. To reach this end, we collected forecasts for key macroeconomic variables from the Bloomberg database and compared them with actual values. To calculate the surprise variables,^5^ we adopted the method used by, among others, Kilponen et al. (2015), whose general formula is presented below:

A.1 $$SURPRISE\_VAR^{\_} c = \, \left( {AV\_VAR\_c \, - \, FV\_VAR\_c} \right)/SD\_VAR\_c.$$

In Equation (A.1), *SURPRISE_VAR*^*_*^*c* denotes the level of surprise of the variable *VAR* for country *c*. *AV_VAR_c* is the actual value of the variable *VAR* for country *c*. *FV_VAR_c* is the value of forecast (constructed on the basis of analysts’ forecasts) for the variable *VAR* for country *c*. *FV_VAR_c* is the standard deviation of the variable (*AV_VAR_c − FV_VAR_c*). The following macroeconomic categories were included in the analysis:

*VAR* = *GDP* in the case of gross domestic product growth.

*VAR* = *RS* in the case of the value of retail sales.

*VAR* = *CPI* in the case of the inflation rate.

*VAR* = *UNEMP* in the case of the unemployment rate.

*VAR* = *CA* in the case of the current account balance.

In the empirical investigation, it was assumed that the initiatives undertaken at the individual country’s level exerted only a domestic impact—that is, there were no international spillover effects from monetary policies of the CNB, MNB or NBP to the other CEE-3 countries. Similar assumptions were introduced for macroeconomic surprises. As a result, null restrictions were imposed.

Tables 10, 11 and 12 present the estimates of the parameters for variables associated with anticrisis measures of domestic national banks as well as macroeconomic surprises in mean and volatility equations.

**Table 10:** Estimates of parameters for variables associated with the measures of national banks and macroeconomic surprises in equations explaining means and volatilities in the currency, bond and stock markets of Poland

Window variable	Currency market				Bond market				Stock market			
	1	3	6	11	1	3	6	11	1	3	6	11
Mean equation												
* PL_INTRATE-DOWN*	–	–	–	–	–	− 0.012*	− 0.005*	− 0.004*	–	–	–	0.001**
* PL_INTRATE-UP*	–	–	–	–	–	–	–	–	–	− 0.001*	− 0.001*	–
* PL-INTERVENTION_PUR*	0.008***	0.005***	0.003**	0.002*	–	–	–	–	–	–	–	–
* PL-INTERVENTION_SEL*	− 0.004*	–	–	–	–	–	–	–	–	–	–	–
* SURPRISE_CA_PL*	–	− 0.001*	− 0.001*	− 0.001***	–	–	–	–	–	–	–	–
* SURPRISE_CPI_PL*	–	–	–	–	–	0.007***	0.004***	0.003**	–	–	–	–
* SURPRISE_GDP_PL*	− 0.001**	− 0.001**	− 0.001*	− 0.001*	–	–	–	–	–	–	–	–
* SURPRISE_RS_PL*	–	− 0.001**	− 0.001*	–	–	–	–	–	–	–	–	–
Volatility equation												
* PL_INTRATE-DOWN*	0.002**	0.002***	0.002***	0.002***	0.002**	0.002**	0.002***	0.002***	–	–	0.003***	0.002***
* PL_INTRATE-UP*	− 0.001***	− 0.002***	− 0.002***	− 0.002***	− 0.002***	− 0.002***	− 0.002***	− 0.001***	–	–	–	–
* SURPRISE_CA_PL*	0.001*	0.001***	0.001***	0.001***	–	0.001*	0.001***	0.001***	–	–	–	–
* SURPRISE_CPI_PL*	0.001*	0.001***	0.001***	0.001***	–	− 0.001*	–	–	0.001*	0.001*	0.001**	0.001**
* SURPRISE_GDP_PL*	− 0.001*	− 0.001***	− 0.001***	− 0.001***	–	–	–	–	–	–	0.001**	0.001***
* SURPRISE_RS_PL*	–	–	–	–	–	0.001*	0.001*	0.001**	–	–	–	–

Source: Authors’ own calculations.

*, ** and *** indicate statistical significance at the 0.1, 0.05 and 0.01 levels, respectively. The symbol ‘-’ denotes statistical insignificance.

**Table 11:** Estimates of parameters for variables associated with the measures of national banks and macroeconomic surprises in equations explaining means and volatilities in the currency, bond and stock markets of Hungary

Window Variable	Currency market				Bond market				Stock market			
	1	3	6	11	1	3	6	11	1	3	6	11
Mean equation												
*FGS_ANNOUNCEMENT*	–	–	–	–	− 0.102**	–	–	–	0.012*	0.005*	0.004*	0.003*
*FGS_START*	− 0.009**	–	–	–	–	–	–	–	–	–	0.004*	–
*FGS_END*	–	–	–	–	–	–	–	–	− 0.013*	–	–	–
*MBPP_ANNOUNCEMENT*	0.004**	–	–	–	–	–	–	–	–	–	–	− 0.005**
*MBPP_DETAILS*	–	–	− 0.001*	− 0.001*	–	–	–	–	–	–	–	–
*MBPP-END_ ANNOUNCEMENT*	–	–	–	–	–	–	–	–	0.010*	–	–	–
*SFP_END*	–	–	–	–	–	–	–	–	0.014*	–	–	–
*HUCBPP_ANNOUNCEMENT*	0.008***	0.004***	0.003***	0.002***	–	–	–	–	–	–	–	–
*HU_INTRATE-DOWN*	–	–	0.001***	0.001**	–	–	–	–	–	–	–	0.001*
*HU_INTRATE-UP*	–	–	–	–	–	–	–	–	− 0.008**	–	–	–
*SURPRISE_CPI_HU*	–	–	–	0.001*	0.012**	0.010***	0.009***	0.007***	–	–	–	–
*SURPRISE_GDP_HU*	–	–	− 0.001*	− 0.001*	–	− 0.005*	–	–	0.003**	0.002***	0.001*	–
*SURPRISE_RS_HU*	− 0.001*	–	–	–	–	–	–	–	–	–	–	–
*SURPRISE_UNEMP_HU*	–	–	–	–	–	–	–	–	–	− 0.001*	–	–
Volatility equation												
*FGS_ANNOUNCEMENT*	− 0.004***	− 0.005***	− 0.004***	− 0.003***	− 0.006***	− 0.006***	− 0.002*	− 0.002*	− 0.006***	− 0.004***	− 0.004***	− 0.003***
*FGS_START*	0.010***	0.011***	0.009***	0.008***	0.008***	0.008***	0.009***	0.012***	–	–	–	–
*FGS_END*	–	–	–	–	0.003***	–	–	–	0.006***	0.005***	0.005***	0.004***
*MBPP_ANNOUNCEMENT*	0.002***	0.004***	0.003***	0.003***	0.006***	0.006***	0.007***	0.006***	0.010***	0.008***	0.008***	0.007***
*MBPP_DETAILS*	0.005***	0.008***	0.007***	0.006***	0.007***	0.007***	0.006***	0.006***	0.011***	0.010***	0.009***	0.009***
*MBPP-END_ ANNOUNCEMENT*	− 0.008***	− 0.007***	− 0.006***	− 0.005***	− 0.012***	− 0.012***	− 0.012***	− 0.011***	− 0.011***	− 0.010***	− 0.009***	− 0.009***
*SFP_END*	− 0.026***	− 0.019***	− 0.016***	− 0.015***	− 0.013***	− 0.010***	− 0.008***	− 0.006***	− 0.006***	− 0.006***	− 0.005***	− 0.004**
*HUCBPP_ANNOUNCEMENT*	–	–	–	–	0.008***	0.007***	0.008***	0.009***	0.004***	0.004***	0.004***	0.003***
*HU_INTRATE-UP*	0.020***	0.018***	0.017***	0.016***	0.020*	0.019***	0.016***	0.014***	–	–	–	–
*SURPRISE_UNEMP_HU*	–	–	–	–	–	0.002**	0.002***	0.002***	–	–	–	–

Source: Authors’ own calculations.

*, ** and *** indicate statistical significance at the 0.1, 0.05 and 0.01 levels, respectively. The symbol ‘-’ denotes statistical insignificance.

**Table 12:** Estimates of parameters for variables associated with the measures of national banks and macroeconomic surprises in equations explaining means and volatilities in the currency, bond and stock markets of the Czech Republic

Window Variable	Currency market				Bond market				Stock market			
	1	3	6	11	1	3	6	11	1	3	6	11
Mean equation												
*CZ_INTRATE-DOWN*	0.004**	0.002**	0.001**	–	− 0.018*	− 0.011*	− 0.006*	− 0.003*	0.004*	0.004**	0.001*	–
*CZ_INTRATE-UP*	− 0.001*	–	–	–	–	–	–	–	–	–	–	–
*SURPRISE_GDP_CZ*	–	–	–	–	–	–	–	–	0.001*	–	–	–
*SURPRISE_RS_CZ*	–	–	–	–	–	–	–	–	–	0.001***	0.001*	–
*SURPRISE_UNEMP_CZ*	–	–	–	–	0.005*	0.002*	0.001*	0.001*	–	–	–	–
Volatility equation												
*CZ_INTRATE-UP*	–	–	–	–	–	–	–	–	0.011***	0.010***	0.008***	0.006***
*ERC*	− 0.001***	− 0.001***	− 0.001***	− 0.001***	–	–	–	–	–	–	–	–
*SURPRISE_CPI_CZ*	–	0.001**	0.001**	–	–	–	–	–	–	–	–	0.001**
*SURPRISE_GDP_CZ*	–	–	–	–	0.001*	0.002**	0.002**	0.001**	–	–	–	0.001**
*SURPRISE_RS_CZ*	–	–	–	–	–	–	–	–	–	–	–	− 0.002**
*SURPRISE_UNEMP_CZ*	0.001***	0.001***	0.001***	0.001***	–	–	–	–	–	–	–	–

Source: Authors’ own calculations.

*, ** and *** indicate statistical significance at the 0.1, 0.05 and 0.01 levels, respectively. The symbol ‘-’ denotes statistical insignificance.

## Appendix 2

Following the estimation of the parameters of the VAR-AGDCC-GARCH model, impulse response functions were calculated. Figures 1, 2 and 3 present results of the impulse-response analysis within markets and between countries, while Figs. 4, 5 and 6 present the results of the impulse-response analysis within countries and between markets.

## Footnotes

1 Conventional monetary policy easing through interest rate cuts, which we also included in this study, was mainly conducted by the ECB in the years 2008 and 2009 and, later, in the second part of 2013 onward. This study does not account for the measures undertaken by the ECB to mitigate the impact of the coronavirus disease 2019 pandemic on the euro-area economy.

2 The use of global VARs could be considered as a useful alternative (Pesaran et al. 2004; Dees et al. 2007; Burriel and Galesi 2018).

3 Estimates of parameters for control variables are presented in Appendix 1.
